# Acute Mitral Papillary Muscle Rupture Secondary to Cardiac Amyloidosis

**DOI:** 10.1016/j.atssr.2024.09.020

**Published:** 2024-10-16

**Authors:** Takashi Nagase, Noriyuki Kashiyama, Natsumi Horikawa, Nozomu Sakai, Masahiro Ryugo, Osamu Monta, Yasushi Tsutsumi, Shinichiro Oda

**Affiliations:** 1 Department of Cardiovascular Surgery, Fukui Cardiovascular Center, Fukui, Japan; 2 Department of Cardiovascular Surgery, Kyoto Prefectural University of Medicine, Kyoto, Japan

## Abstract

Acute mitral valve regurgitation due to papillary muscle rupture secondary to cardiac amyloidosis is rare. A 75-year-old woman without prior heart disease or chest trauma presented with acute mitral regurgitation and shock. Coronary angiography demonstrated patent coronary arteries. Emergency surgery revealed a prolapse of the anterior leaflet with posteromedial papillary muscle rupture. Mitral valvuloplasty was performed using artificial chordae and an annuloplasty ring. Histopathologic examination of the ruptured papillary muscle confirmed amyloid deposition between the cardiomyocytes. This case underscores the importance of cardiac amyloidosis in the differential diagnosis of patients with papillary muscle rupture without coronary artery stenosis.

Nonischemic mitral valve regurgitation (MR) with papillary muscle rupture is a rare clinical entity. Herein, we present a case of papillary muscle rupture secondary to cardiac amyloidosis (CA).

A 75-year-old woman without prior heart diseases or chest trauma presented to another institution by emergency medical services due to sudden-onset dyspnea, resulting in multiorgan failure that necessitated intubation. The patient was then referred to our institution for further surgical intervention.

Upon arrival at our hospital, the patient was in shock and hemodynamically unstable. Electrocardiography showed sinus rhythm without significant ST-T wave changes (Figure 1A). Routine laboratory results revealed elevated levels of creatine kinase (624 IU/L; normal, <170 IU/L), creatine kinase MB (76.2 ng/mL; normal, <5.0 ng/mL), troponin T (1.465 ng/mL; normal, <0.099 ng/mL), and pro-brain natriuretic peptide (7250 pg/mL; normal, <125 pg/mL). Chest radiography was consistent with acute pulmonary edema.

**Figure 1:**
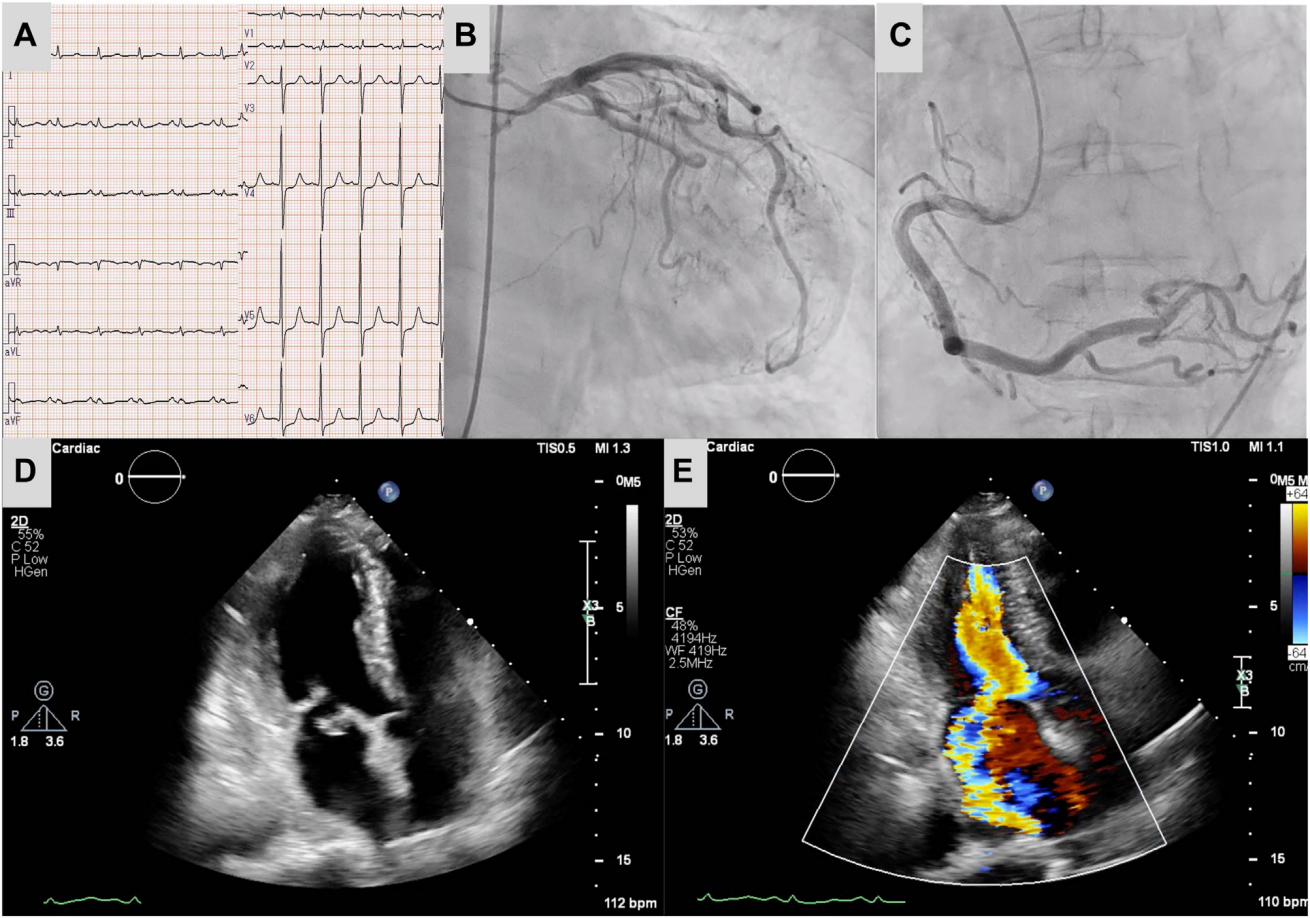
(A) Preoperative electrocardiography shows sinus rhythm without ST-T wave changes. Angiography in the (B) left and (C) right coronary arteries. No obstructive lesions are observed in both coronary arteries. Preoperative transthoracic echocardiography shows (D) anterior leaflet prolapse and (E) severe mitral valve regurgitation.

Although emergency coronary angiography revealed patent coronary arteries (Figures 1B, 1C), echocardiography demonstrated normal ventricular wall motion with severe MR and anterior leaflet prolapse (Figures 1D, 1E). Echocardiography did not reveal amyloidosis features such as hypertrophy or highly echogenic myocardium.

These findings prompted emergency cardiac surgery, and cardiopulmonary bypass was established. After cardiac arrest, right-sided left atriotomy was performed for mitral valve access. Intraoperative examination revealed a ruptured head of the posteromedial papillary muscle with concomitant prolapse of the A2 and A3 anterior leaflet segments, whereas the A1 segment appeared intact. The resected posteromedial papillary muscle (Figure 2A) showed necrotic changes at the rupture site.

**Figure 2:**
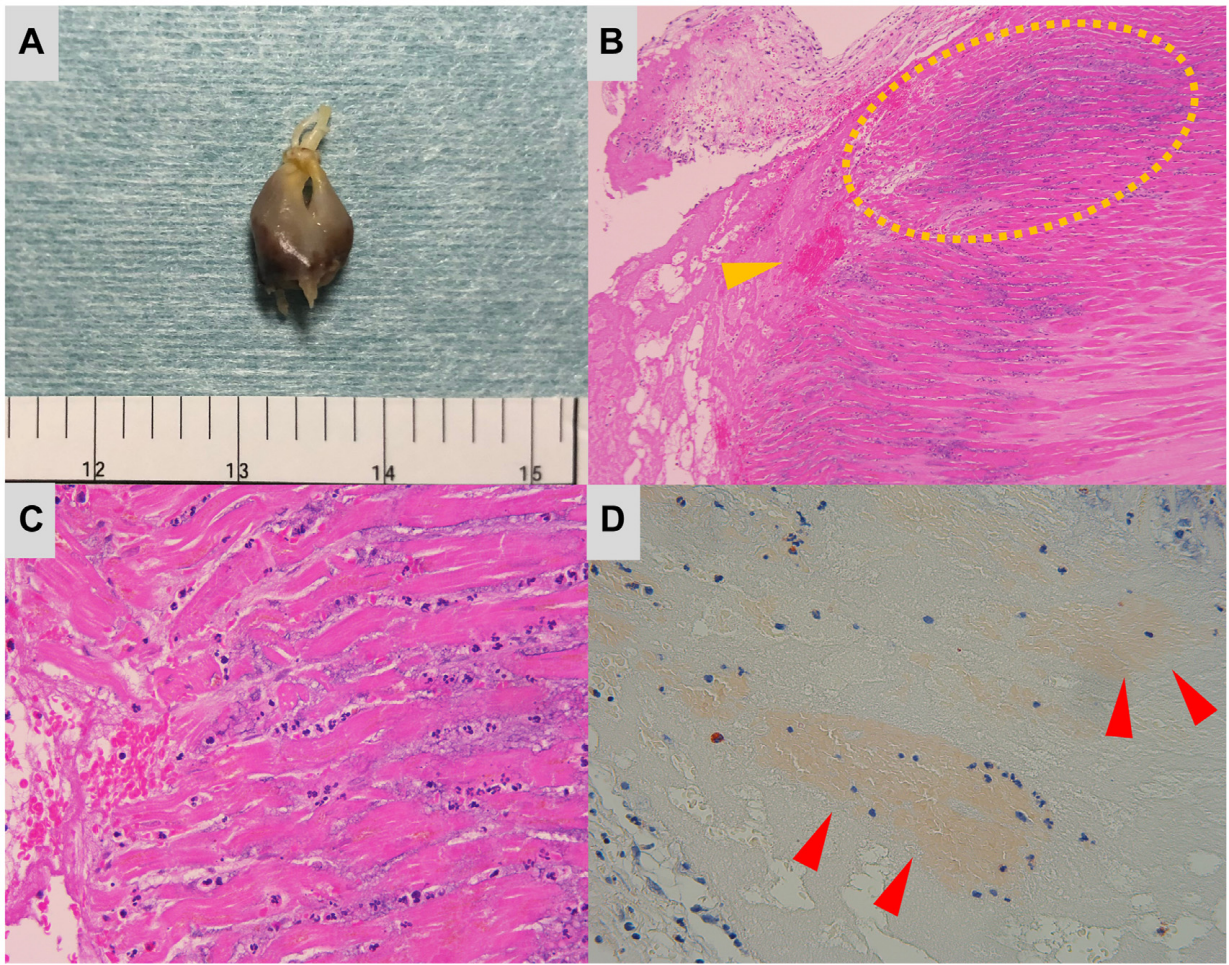
(A) Macroscopic specimen of the ruptured posteromedial papillary muscle. The site of the rupture appears necrotic. (B) Lower-power image shows prominent neutrophil infiltration (yellow dotted line) and focal hemorrhage (yellow arrow) at the site of the papillary muscle rupture. Chronic fibrosis and inflammatory cell infiltration are observed between the cardiomyocytes (original magnification ×10). (C) Higher-power image of the histopathologic findings (original magnification ×40). (D) Congo red staining yielded positive results for eosinophilic amorphous material (red arrows) (original magnification ×40).

Mitral valvuloplasty was subsequently performed with artificial chordae. Expanded polytetrafluoroethylene (Gore-Tex CV sutures; W. L. Gore & Associates, Flagstaff, AZ) were used to reconstruct the prolapsed anterior leaflet. Additionally, a semirigid mitral annuloplasty ring (Memo 3D; LivaNova, London, United Kingdom) was used to optimize valve coaptation. Cardiopulmonary bypass time and aortic cross-clamp time were 107 and 67 minutes, respectively. Postoperative echocardiography demonstrated successful reduction of the MR to mild severity (Figures 3A, 3B).

**Figure 3:**
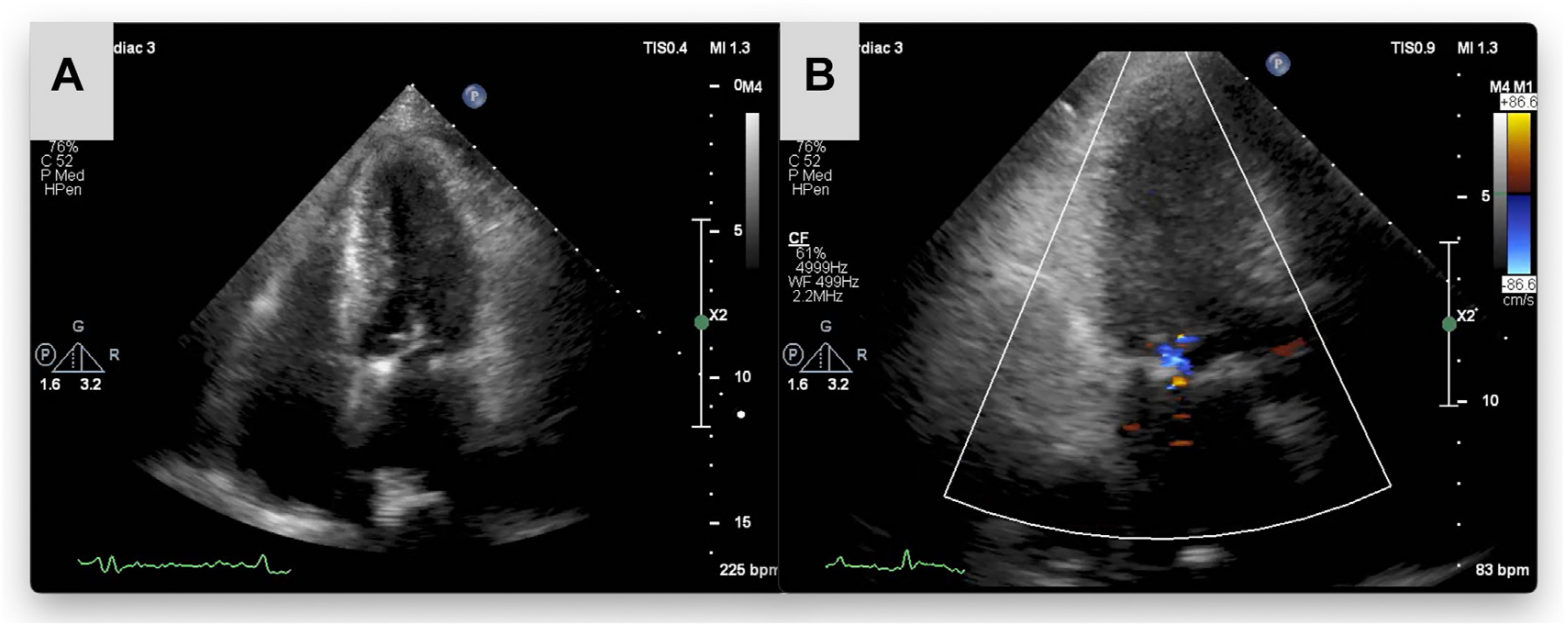
(A) Postoperative transthoracic echocardiography shows repair of the anterior leaflet prolapse. (B) Findings show that the mitral regurgitation is reduced to mild severity.

Histopathologic examination of the ruptured papillary muscle revealed prominent neutrophil infiltration and focal hemorrhage (Figures 2B, 2C). Moreover, chronic fibrosis and inflammatory cell infiltration were observed between the cardiomyocytes. Notably, Congo red staining yielded positive results for eosinophilic amorphous material, exhibiting apple-green birefringence under polarized light (Figure 2D).

Despite successful surgical intervention and extubation 5 days postoperatively, she experienced persistent complications, including altered mental status, renal dysfunction, and hematochezia. Unfortunately, the patient died of these complications 23 days postoperatively. We could not perform an autopsy owing to the lack of consent from the patient’s family.

## Comment

Although myocardial ischemia remains the most common cause of MR with papillary muscle rupture, nonischemic etiologies are also possible but are uncommon. These include infective endocarditis, blunt chest trauma, complications of invasive procedures, and idiopathic presentations.^1^ Among these etiologies, papillary muscle rupture due to CA is extremely rare, with only a limited number of published case reports.^2^

CA is typically diagnosed in its advanced stages between ages 70 and 75 years.^3^ Pathologically, it arises from the progressive deposition of insoluble amyloid fibrils within the myocardium, leading to a spectrum of cardiac dysfunction.^3^ These deposits can also be found in coronary arteries, potentially causing symptomatic ischemic heart disease.^2^ Roberts and colleagues^4^ reported that the autopsy results of 54 patients with CA revealed intramural amyloid deposits in the coronary arteries of all cases. However, Mueller and colleagues^5^ emphasized the difficulty in diagnosing ischemic heart disease from obstructive intramural coronary amyloidosis before death or after cardiac transplantation. This suggests that some cases of idiopathic papillary muscle rupture might have been misdiagnosed and could potentially be linked to amyloidosis.

In this case, the patient lacked any clinical history or findings suggestive of CA preoperatively. In fact, her diagnosis was made after a comprehensive histopathologic examination. We therefore postulate that focal ischemia due to intramural coronary artery obstruction from CA ultimately led to papillary muscle rupture.

The surgical strategy for papillary muscle rupture remains controversial. Although several reports^6^^,^^7^ on ischemic papillary muscle rupture indicated that more patients underwent mitral valve replacement than mitral valvuloplasty, none commented on the superiority of either surgical method.

Mitral transcatheter edge-to-edge repair (MTEER) is another treatment strategy for patients with cardiogenic shock^8^; however, our institution is not equipped to perform emergency MTEER. Considering the urgency in this case, a thorough assessment of the mitral valve anatomy was not feasible; hence, MTEER was not considered. Furthermore, we determined that successful surgical mitral repair was achievable based on the echocardiographic findings.

In this case, MR was caused by a partial rupture of the papillary muscle, with a sufficient amount of normal papillary muscle tissue remaining intact. The cause of the regurgitation was relatively straightforward, thereby allowing the repair to be successfully completed in a short amount of time. Considering that CA was the cause of the MR, there is a possibility of recurrent papillary muscle rupture due to myocardial fragility underlying CA. Therefore, mitral valve replacement might be a better option in cases of papillary muscle rupture with an unknown cause.

In conclusion, this study describes a rare case of papillary muscle rupture secondary to CA. Our findings underscore the importance of considering CA in the differential diagnosis for patients with papillary muscle rupture without coronary artery stenosis. Accurate diagnosis will influence future treatment strategies and patient management for such cases.

## Declaration of Generative AI and AI-Assisted Technologies in the Writing Process

During the preparation of this work, the authors used ChatGPT for proofreading the English writing. After using this tool, the authors reviewed and edited the content as needed and take full responsibility for the content of the publication.
